# Retraction: Vitamin D sufficiency, a serum 25-hydroxyvitamin D at least 30 ng/mL reduced risk for adverse clinical outcomes in patients with COVID-19 infection

**DOI:** 10.1371/journal.pone.0305303

**Published:** 2024-06-06

**Authors:** The PLOS ONE Editors

Following the publication of this article [1], concerns were raised about the validity of results and conclusions reported in the article and about undisclosed competing interests, as described in a previous Expression of Concern [2]. *PLOS ONE* has now concluded its investigation into these concerns.

Our editorial assessment involved input from a statistical reviewer and members of the *PLOS ONE* Editorial Board. Having considered their feedback and the authors’ responses in detail, we have found that 1) the reported study design, including inclusion criteria and statistical analysis methods, was not sufficiently robust to address the research question, and 2) the methodology was not reported in sufficient detail to enable reproduction of this study. As such, we have concluded that the article’s conclusions are not supported by the reported data.

In light of these concerns, the *PLOS ONE* Editors retract this article. We regret that these issues were not identified and addressed prior to the article’s publication.

ZM, MAS, and MFH did not agree with the retraction. ME, MP, SK, HMT, AH, MM, MN, and AS either did not respond directly or could not be reached.
